# Staging Paradox and recurrence pattern among stage IIB, IIC, and IIIA Colon cancers: a retrospective cohort study

**DOI:** 10.1007/s00384-024-04737-1

**Published:** 2024-10-14

**Authors:** Yu-Tso Liao, John Huang, Ji-Shiang Hung, Kai-Wen Huang, Jin-Tung Liang

**Affiliations:** 1 Graduate Institute of Clinical Medicine, College of Medicine, National Taiwan University, Taipei, Taiwan; 2 Division of Colorectal Surgery, Department of Surgery, National Taiwan University Hospital, Hsin-Chu Branch, Hsinchu, Taiwan; 3 Division of Colorectal Surgery, Department of Surgery, National Taiwan University Hospital and College of Medicine, Taipei, Taiwan

**Keywords:** Colon cancer, Staging paradox, Locoregional recurrence, Stage IIB/C, Stage IIIA, Chemotherapy

## Abstract

**Purpose:**

The survival rates of patients with stage IIB and IIC colon cancer are paradoxically inferior to that of patients with stage IIIA colon cancer. This study aimed to examine the oncological outcomes and investigate the factors that could affect the staging paradox among stage IIB, IIC, and IIIA colon cancers based on a 9-year cancer database.

**Methods:**

Patients with stage IIB (pT4aN0M0), IIC (pT4bN0M0), or IIIA (pT1-2N1M0) colon cancer were retrospectively selected from a prospectively maintained medical database from January 2011 to December 2019. Factors that might influence the staging paradox, including radicality, harvested lymph nodes, and chemotherapy administration, were examined.

**Results:**

A total of 282 patients (stage IIB, *n* = 59; stage IIC, *n* = 46; and stage IIIA, *n* = 177) were enrolled. Patients with stage IIB/C cancer demonstrated higher carcinoembryonic antigen levels, larger tumor size, more frequent tumor obstruction, and higher locoregional recurrence than those with stage IIIA cancer. With respect to 10-year locoregional recurrence-free survival and cancer-specific survival, patients with stage IIB and IIC cancers had significantly lower survival rates than did those with stage IIIA cancer (73.7% vs. 66.3% vs. 91.2%, *P* = 0.0003; 5.4% vs. 10.9% vs. 11.2%, *P* = 0.0023). The staging paradox persisted in patients who underwent R0 resection, had harvested lymph nodes ≥ 12, and received chemotherapy, as confirmed by multivariate regression analysis.

**Conclusions:**

Based on the inferior oncological outcomes and higher locoregional recurrence rate, this study highlighted the need for intensified cytotoxic chemotherapy specific to this recurrence pattern for patients with stage IIB/C colon cancer.

## Introduction

In the tumor-node-metastasis (TNM) system for colorectal cancer, nodal status is a significantly more important indicator of prognosis than the depth of invasion. A positive nodal status is considered a systemic disease status, having a worse oncological outcome and requiring systemic chemotherapy to prolong survival. In contrast, a negative nodal status is considered a local disease status, mainly relying on R0 surgical resection. Conventionally, systemic chemotherapy for negative nodal status is unnecessary, unless high-risk features, such as pT4, are present [1, 2]. Nevertheless, patients with stage IIB/C colon cancer (T4aN0M0 and T4bN0M0) have worse oncological outcomes than do those with stage IIIA colon cancer (T1-2N1 and T1N2a) [3, 4]. The discrepancy in survival between the TNM system and real-world data is referred to as the “staging paradox,” implying a mismatch between TNM staging and its relevant survival.

Possible explanations to reconcile the staging paradox and TNM system include staging migration due to inadequate lymph node harvest, a higher positive surgical margin for T4N0, lack of chemotherapy for stage IIB/C, and the aggressive biological characteristics of stage IIB/C [4]. Multivisceral resection is required for T4b colorectal cancer to achieve negative margins [5]. A higher percentage of positive surgical margins in pT4N0M0 colon cancer may contribute to worse oncological outcomes in stage IIB/C than in stage IIIA [6]. Furthermore, inadequate lymph node retrieval for stage IIB/C leads to misclassification of pT4N1 as pT4N0 [4]. However, there is little evidence to support these hypotheses, and some have been challenged [7, 8].

The effectiveness of chemotherapy has long been considered uncertain, considering its potential adverse effects and limited survival benefits in patients with stage IIB/C colon cancer [2, 9]. The lack of systemic adjuvant chemotherapy for patients with stage IIB/C colon cancer may be attributed to the staging paradox. However, real-world data are not as robust and may yield contradictory results [7, 8, 10, 11].

Finally, aggressive cancer behavior has been suggested in stage IIB/C. Molecular differences seem to exist between stage IIB/C and IIIA colon cancers; however, only a few studies have reported this finding [12].

We hypothesize that the staging paradox is universal. Furthermore, the identification of relevant factors other than radicality, harvested lymph node, and conventional chemotherapy administration in patients with stage IIB/C colon cancer is paramount for tailoring specific chemotherapies to improve survival outcomes. This study aimed to investigate the oncological outcomes of stage IIB, IIC, and IIIA colon cancers with respect to radicality, harvested lymph nodes, and chemotherapy administration and to evaluate factors that could affect the staging paradox among stage IIB, IIC, and IIIA colon cancers based on a 9-year cancer database.

## Methods

### Patient selection

Patients with stage IIB (pT4aN0M0), IIC (pT4bN0M0), or IIIA (pT1-2N1M0, pT1N2aM0) colon cancer were retrospectively selected from a prospectively maintained database (Integrative Medical Database, National Taiwan University Hospital [NTUH]) from January 2011 to December 2019. The International Classification of Diseases (ICD) 10 (ICD9) codes were C183 (1530), C184 (1531), C186 (1532), C187 (1533), C180 (1534), C181 (1535), C182 (1536), C185 (1537), C188 (1538), C189 (1539), C19 (1540), and C20 (1541). Clinicopathological data were retrieved from a database that included patients treated during the 9-year period at the NTUH and NTUH–Hsinchu Branch, a branch hospital of the NTUH. Colon cancer was staged according to the 8th edition of the American Joint Committee on Cancer (AJCC) staging system [13]. The classification remains unchanged throughout the 7th and 8th editions of the AJCC TNM staging system, with pT4a defined as the maximal invasion depth penetrating but confined to the serosa and pT4b defined as the invasion depth penetrating and invading the adjacent organ, as confirmed by a pathologist. This study was approved by the Institutional Review Board of the NTUH–Hsinchu Branch (202111067RINB), and the requirement for written informed consent from each participant was waived owing to the retrospective nature of this study. This study adhered to the Strengthening the Reporting of Observational Studies in Epidemiology guidelines.

### Inclusion and exclusion criteria

The inclusion criteria were as follows: (1) pathologically proven colon adenocarcinoma; (2) pathological stage IIB (pT4aN0M0), IIC (pT4bN0M0), and IIIA (pT1-2N1M0, pT1N2aM0); and (3) tumor location above the peritoneal reflection. The exclusion criteria were as follows: (1) pathologies other than adenocarcinoma, such as squamous cell carcinoma and neuroendocrine tumors; (2) presence of synchronous colorectal cancer; (3) presence of distant metastasis; (4) tumor location below the peritoneal reflection; and (5) missing clinicopathological data that would interfere with the analysis of survival outcomes. This study included patients who underwent elective or emergency surgery.

The right colon included the cecum, ascending colon, hepatic flexure, and proximal transverse colon, whereas the left colon included the distal transverse colon, splenic flexure, descending colon, and sigmoid colon. Clinical obstruction was defined as (1) symptomatic obstruction requiring refraining from oral intake and intravenous fluid administration, (2) presence of luminal narrowing upon colonoscopy, or (3) presence of bowel obstruction proximal to the tumor on abdominal plain film or computed tomography. Anastomotic leakage was defined as the presence of bowel contents within the drainage tube. Locoregional recurrence was defined as the presence of a tumor adjacent to a previous surgical site on radiological imaging. Distant metastasis was defined as the presence of a tumor in distant organs, such as the liver, lung, and retroperitoneum.

Adjuvant chemotherapy was administered according to the relevant National Comprehensive Cancer Network (NCCN) guidelines for the year the patient was being treated. The adjuvant chemotherapy regimens included uracil–tegafur (UFUR^®^), capecitabine (Xeloda^®^), 5-fluorouracil, oxaliplatin, and their combination. Oncological outcomes were evaluated based on locoregional recurrence-free survival (defined as the time from diagnosis to locoregional recurrence) and cancer-specific survival (defined as the time from diagnosis to colon cancer-related death). To evaluate the oncological outcomes of real-world data, 10-year oncological outcomes, including 10-year locoregional recurrence-free survival and cancer-specific survival, were used [3, 6, 7, 10, 12, 14]. We used cancer-specific death as an event while performing the survival analysis, primarily to exclude non-cancer-related deaths during a follow-up period of 10 years.

### Statistical analyses

Categorical variables in patients with stage IIB, IIC, and IIIA colon cancer were analyzed using the chi-squared and Fisher’s exact tests, whereas continuous variables were compared among the three patient groups using one-way analysis of variance. Survival probabilities were evaluated using the Kaplan–Meier survival analysis, whereas risk factors influencing oncological outcomes were examined using the log-rank test. Univariate and multivariate Cox proportional hazards analyses were performed to evaluate the hazard ratios and 95% confidence intervals among patients with stage IIB, IIC, and IIIA colon cancers. All analyses were performed using Small Stata statistical software version 13.0 for Windows 11, with statistical significance set at a two-sided *P*-value < 0.05.

## Results

**Fig. 1:**
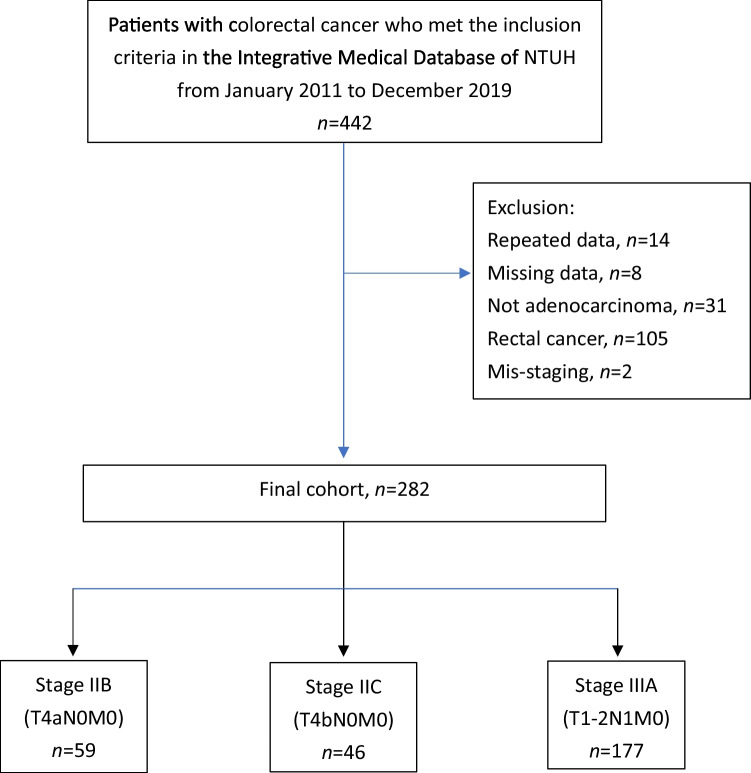
Flowchart of patient selection for this study

A total of 282 patients were included in this study. Among these patients, 59, 46, and 177 had stage IIB, IIC, and IIIA cancers, respectively. Figure 1 illustrates a flowchart of the patient selection process.

Table 1 summarizes the clinical and surgical characteristics of patients. Patients with stage IIB cancer were significantly older than those with stage IIIA cancer (*P* = 0.0037). Carcinoembryonic antigen (CEA) levels were significantly higher in patients with stage IIB and IIC cancers (*P* < 0.0001 for both) than in those with stage IIIA cancer. Clinical obstruction was more frequent in patients with stage IIB and IIC cancers (*P* < 0.0001) than in those with stage IIIA cancer. Patients with stage IIB/C colon cancer were more likely to undergo stoma or stenting to relieve clinical obstruction than were those with stage IIIA colon cancer (*P* < 0.0001 and *P* = 0.0222, respectively).

**Table 1:** Clinical and surgical features of patients with stage IIB, IIC, and IIIA colon cancers

	Stage IIB (*n* = 59)	Stage IIC (*n* = 46)	Stage IIIA (*n* = 177)	*P*-value
Age, mean ± SD	64.5 ± 7.1	69.3 ± 12.8	63.5 ± 11.24	0.0056^a^
Sex, male/female, *n*	33 /26	23 /23	82 /95	0.4364
Tumor location, *n* (%)				0.1731
Right-sided colon	27 (45.8)	13 (28.3)	64 (36.2)	
Left-sided colon	32 (54.2)	33 (71.7)	113 (63.8)	
CEA, *n* (%)				< 0.0001^b^
< 5	43 (72.9)	30 (65.2)	171 (96.7)	
≥ 5	16 (27.1)	16 (37.8)	6 (3.4)	
ASA, *n* (%)				0.0107^c^
I	30 (50.9)	19 (41.3)	100 (56.5)	
II	16 (27.1)	9 (19.6)	52 (29.4)	
III	12 (20.3)	18 (39.1)	24 (13.6)	
IV	1 (1.7)	0 (0.0)	1 (0.6)	
Emergency surgery, *n* (%)	1 (1.7)	1 (2.2)	0 (0.0)	0.1755
Surgical method, *n* (%)				0.0016 ^d^
Open surgery	20 (33.9)	29 (63.0)	29 (16.4)	
Laparoscopic/robotic surgery	39 (66.1)	17 (37.0)	148 (83.6)	
Clinical obstruction, *n* (%)	33 (55)	26 (56.5)	0 (0.0)	< 0.0001^e^
Requiring stoma	6 (6.7)	4 (8.7)	0 (0.0)	0.0001^f^
Requiring stenting	2 (3.3)	0 (0.0)	0 (0.0)	0.0222^g^

^a^Post-hoc test showed stage IIB vs. stage IIIA, *P* = 0.0037.^b^Post-hoc test showed stage IIB vs. stage IIIA, *P* < 0.0001 and stage IIC vs. stage IIIA, *P* < 0.0001.^c^Post-hoc test showed stage IIC vs. stage IIIA, *P* = 0.0013.^d^Post-hoc test showed stage IIB vs. stage IIIA, *P* = 0.0002 and stage IIC vs. stage IIIA, *P* < 0.0001.^e^Post-hoc test showed stage IIB vs. stage IIIA, *P* < 0.0001 and stage IIC vs. stage IIIA, *P* < 0.0001.^f^Post-hoc test showed stage IIB vs. stage IIIA, *P* < 0.0001 and stage IIC vs. stage IIIA, *P* < 0.0001.^g^Post-hoc test showed stage IIB vs. stage IIIA, *P* = 0.0139.*SD* standard deviation, *CEA* carcinoembryonic antigenOnly significant *P*-values are shown for the post-hoc test.

Table 2 presents the pathological and oncological outcomes of patients. The R0 rate was significantly lower in patients with stage IIB and IIC cancers than in those with stage IIIA cancer (*P* = 0.0002 and *P* < 0.0001, respectively). The perineural invasion rate was higher in patients with stage IIC and IIIA cancers than in those with stage IIB cancer (*P* = 0.0454 and *P* < 0.0001, respectively). Tumor size was significantly larger in patients with stage IIB and IIC cancers than in those with stage IIIA cancer (*P* = 0.0004 and *P* < 0.0001, respectively). Chemotherapy and oxaliplatin-based chemotherapy rates were significantly lower in patients with stage IIB and IIC cancers than in those with stage IIIA cancer (*P* < 0.0001 and *P* = 0.0002, respectively).

**Table 2:** Pathological and oncological features of patients with stage IIB, IIC, and IIIA colon cancers

	Stage IIB (*n* = 59)	Stage IIC (*n* = 46)	Stage IIIA (*n* = 177)	*P*-value
Radicality, *n* (%)				< 0.0001^a^
R0	52 (88.1)	39 (84.8)	175 (98.9)	
R1/R2	7 (11.9)	7 (15.2)	2 (1.1)	
Tumor differentiation, *n* (%)				0.0860
Grade I–II	50 (84.8)	40 (87.0)	160 (90.4)	
Grade II–III	7 (11.9)	4 (8.7)	8 (4.5)	
Mucinous adenocarcinoma	2 (3.4)	2 (4.4)	1 (0.6)	
Primary tumor removed	0 (0.0)	0 (0.0)	8 (4.5)	
Lymphovascular invasion, *n* (%)				0.7890
Absent	41 (69.5)	32 (70.0)	116 (65.5)	
Present	18 (30.5)	14 (30.0)	61 (34.4)	
Perineural invasion, *n* (%)				0.0082^b^
Absent	40 (67.8)	39 (84.8)	157 (88.7)	
Present	19 (32.2)	7 (15.2)	20 (12.3)	
No. of harvested lymph nodes, *n* (%)				0.2624
≥ 12	56 (94.9)	40 (87.0)	155 (87.6)	
< 12	3 (5.1)	6 (13.0)	22 (12.4)	
Maximal tumor size, mean ± SD	5.5 ± 2.8	7.4 ± 4.0	2.6 ± 1.8	< 0.0001^c^
Anastomotic leakage, *n* (%)	1 (1.7)	2 (4.4)	1 (0.6)	0.1513
Chemotherapy, *n* (%)				< 0.0001^d^
Yes, oxaliplatin-based	11 (18.6)	14 (30.4)	113 (63.8)	
Yes, fluorouracil-based	33 (56.0)	22 (47.8)	47 (26.6)	
No	15 (25.4)	10 (21.7)	17 (9.6)	
Recurrence pattern, initial, *n* (%)				< 0.0001^e^
Locoregional recurrence	8 (13.6)	10 (21.7)	1 (0.1)	
Presence of peritoneal carcinomatosis	7 (11.9)	4 (8.7)	0 (0.0)	< 0.0001^f^
Systemic recurrence	2 (3.4)	2 (4.4)	13 (7.3)	
Site of metastasis^g^				
Liver	2 (3.4)	0 (0.0)	5 (2.8)	
Lung	0 (0.0)	2 (4.4)	8 (4.5)	
Bone	0 (0.0)	1 (2.2)	1 (0.1)	
Brain	0 (0.0)	0 (0.0)	1 (0.1)	
Retroperitoneal Lymph node	0 (0.0)	0 (0.0)	2 (1.1)	
Combined	2 (3.4)	0 (0.0)	0 (0.0)	
Presence of peritoneal carcinomatosis	2 (3.4)	0 (0.0)	0 (0.0)	0.0222^h^
Site of metastasis, liver	2 (3.4)	0 (0.0)	0 (0.0)	
Follow-up periods, mean ± SD	62.2 ± 33.1	59.8 ± 40.0	73.3 ± 33.1	0.0160^i^

^a^Post-hoc test showed stage IIB vs. stage IIIA, *P* = 0.0002 and stage IIC vs. stage IIIA, *P* < 0.0001.^b^Post-hoc test showed stage IIB vs. stage IIC, *P* = 0.0454 and stage IIB vs. stage IIIA, *P* < 0.0001.^c^Post-hoc test showed stage IIB vs. stage IIC, *P* = 0.0004; stage IIB vs. stage IIIA, *P* < 0.0001; and stage IIC vs. stage IIIA, *P <* 0.0001.^d^Post-hoc test showed stage IIB vs. stage IIIA, *P <* 0.0001 and stage IIC vs. stage IIIA, *P* = 0.0002.^e^Post-hoc test showed stage IIB vs. stage IIIA, *P* < 0.0001 and stage IIC vs. stage IIIA, *P* < 0.0001.^f^Post-hoc test showed stage IIB vs. stage IIIA, *P* < 0.0001 and stage IIC vs. stage IIIA, *P* < 0.0001.^g^Patients may have more than one site of distant metastasis.^h^Post-hoc test showed stage IIB vs. stage IIIA, *P* = 0.0138.^i^Post-hoc test showed stage IIC vs. stage IIIA, *P* = 0.0473*SD* standard deviationOnly significant *P*-values are shown for the post-hoc test.

Regarding the recurrence pattern, patients with stage IIB and IIC cancers had a significantly higher initial locoregional pattern than those with stage IIIA cancer (both *P* < 0.0001). The incidence rate of peritoneal carcinomatosis was higher in patients with stage IIB/C colon cancer than in those with stage IIIA colon cancer (*P* < 0.0001). Additionally, the incidence rate of peritoneal carcinomatosis was higher in patients with stage IIB colon cancer than in those with stage IIC and IIIA cancers.

**Fig. 2:**
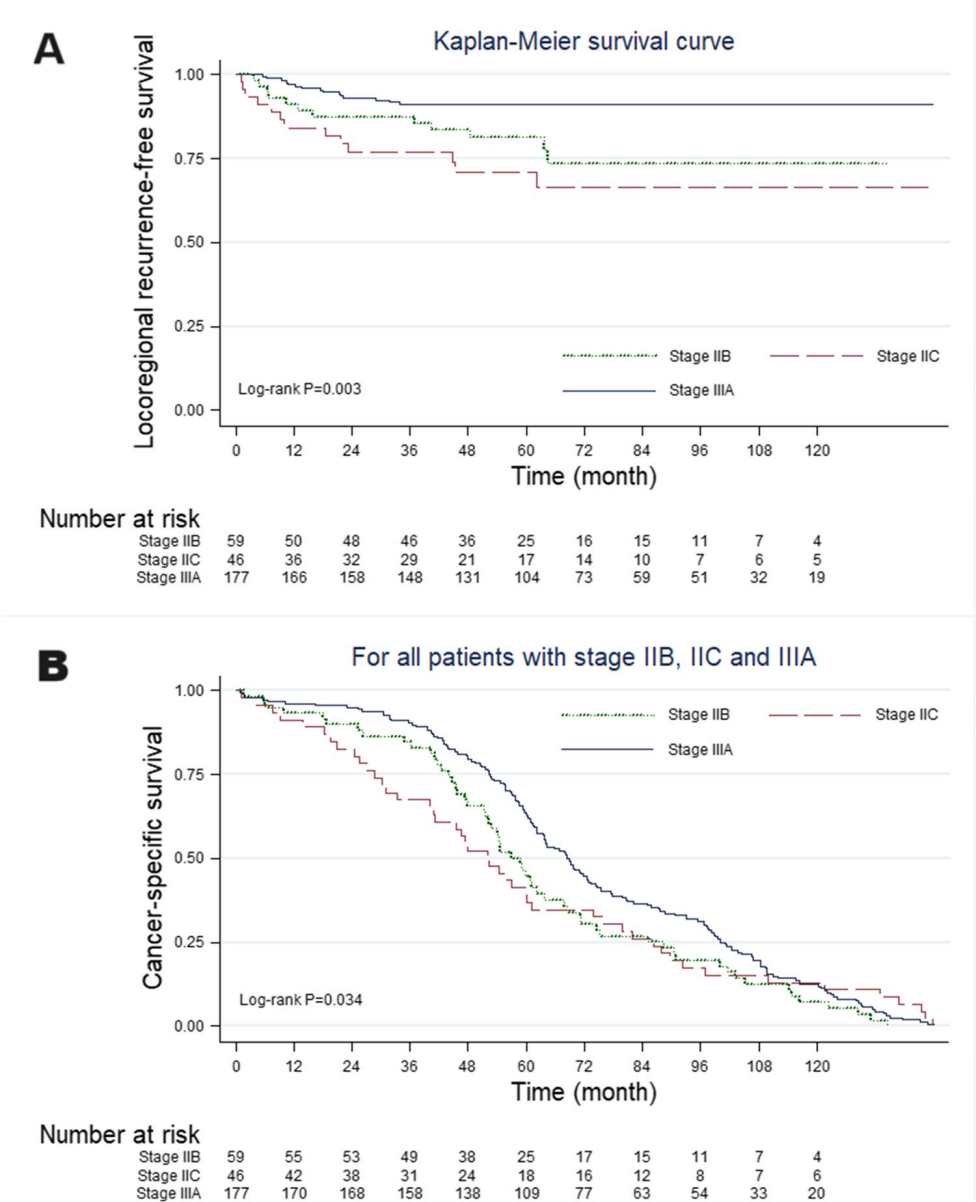
Kaplan–Meier survival curves for patients with stage IIB, IIC, and IIIA colon cancers. (**A**) Locoregional recurrence-free survival. (**B**) Cancer-specific survival

The median follow-up period was 61.9 months. The 10-year locoregional recurrence-free survival rate of patients with stage IIB and IIC cancers was significantly lower than that of patients with stage IIIA cancer (73.7% vs. 66.3% vs. 91.2%, *P* = 0.0003). Additionally, the 10-year cancer-specific survival rate of patients with stage IIB and IIC cancers was significantly lower than that of patients with stage IIIA cancer (5.4% vs. 10.9% vs. 11.2%, *P* = 0.0023) (Fig. 2).

**Fig. 3:**
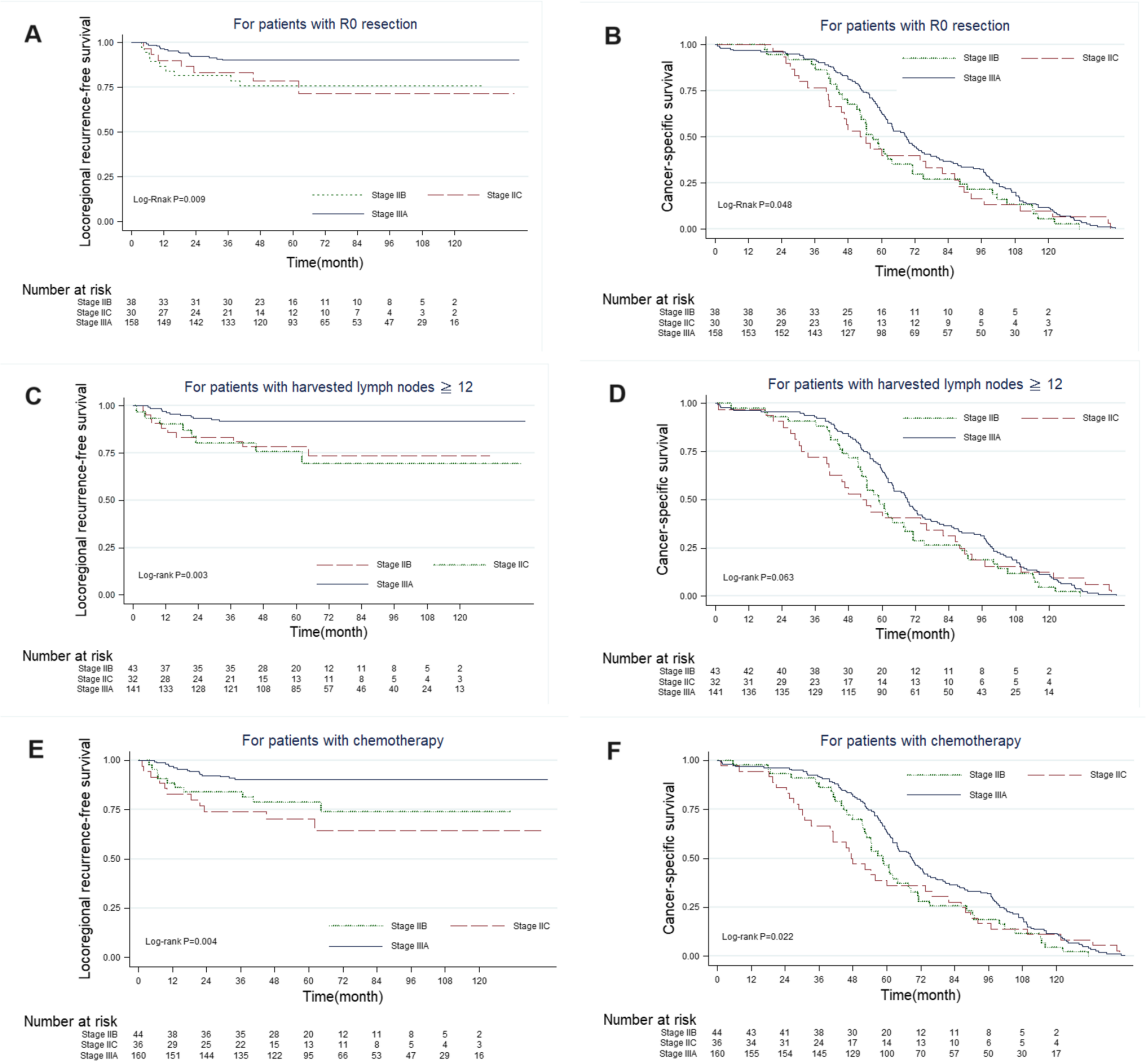
Kaplan–Meier survival curves for patients with stage IIB, IIC, and IIIA colon cancers according to different factors. (**A** and **B**) Patients who underwent R0 resection, (**C** and **D**) patients with lymph node ≥12, and (**E** and **F**) patients who received chemotherapy

Figure 3 presents the 10-year locoregional recurrence-free survival and cancer-specific survival rates according to the factors hypothesized to affect the staging paradox in previous studies. The survival analysis revealed that survival staging persisted for patients who underwent R0 resection, had lymph nodes ≥ 12, and received chemotherapy.

**Fig. 4:**
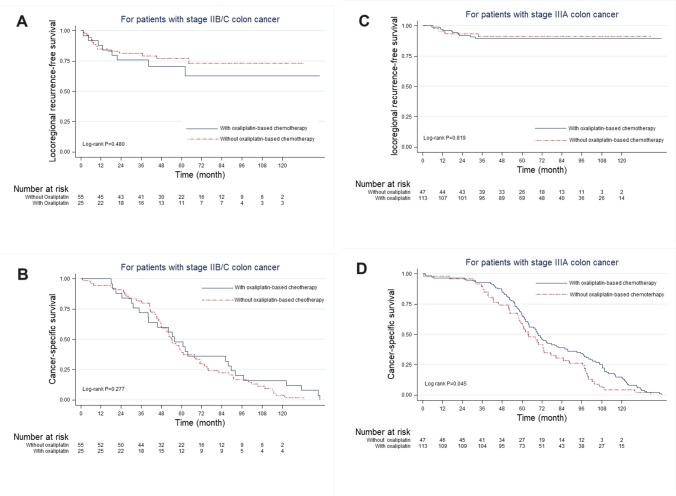
Kaplan–Meier survival curves for patients with stage IIB, IIC, and IIIA colon cancers according to oxaliplatin-based chemotherapy administration. (**A**) Locoregional recurrence-free survival. (**B**) Cancer-specific survival

In patients with stage IIIA colon cancer, the 10-year cancer-specific survival rate improved after adjuvant oxaliplatin-based chemotherapy (4.9% vs. 12.9%, *P* = 0.045). Conversely, the 10-year locoregional recurrence-free survival rate did not improve after adjuvant oxaliplatin-based chemotherapy. Additionally, no survival benefit was observed with oxaliplatin-based chemotherapy in patients with stage IIB or IIC colon cancer (Fig. 4).

Table 3 shows the Cox regression analysis of factors influencing the 10-year locoregional recurrence-free survival and 10-year cancer-specific survival. Univariate regression analysis indicated that stages IIB/C and R1/2 were negatively associated with 10-year locoregional recurrence-free survival. Multivariate regression analysis revealed that stage IIB/C and anastomotic leakage were independent factors negatively associated with 10-year locoregional recurrence-free survival. Regarding 10-year cancer-specific survival, univariate regression analysis revealed that stage IIB/C, age, and anastomotic leakage were negatively associated with 10-year cancer-specific survival. However, multivariate regression analysis demonstrated that age and anastomotic leakage were two independent factors negatively associated with the 10-year cancer-specific survival.

**Table 3:** Cox regression analysis for 10-year locoregional-free survival and cancer-specific survival

	Locoregional-free survival				Cancer-specific survival			
	Univariate		Multivariate		Univariate		Multivariate	
	HR (95% CI)	*P*-value	HR (95% CI)	*P*-value	HR (95% CI)	*P*-value	HR (95% CI)	*P*-value
Stage		0.003		0.008		0.034		0.072
IIB	Ref		Ref		Ref		Ref	
IIC	0.600 (0.427–0.842)		0.593 (0.404–0. 873)		0.854 (0.739–0.989)		0.868 (0.743–1.013)	
IIIA	0.600 (0.427–0.842)		0.593 (0.404–0. 873)				0.868 (0.743–1.013)	
Radicality		0.004		0.089		0.090		0.232
R0	Ref		Ref		Ref		Ref	
R1/2	3.638 (1.527–8.669)		2.285 (0.882–5.918)		1.551 (0.934–2.576)		1.376 (0.815–2.321)	
Lymph nodes		0.176		0.067		0.311		0.433
< 12	Ref		Ref		Ref		Ref	
≥ 12	0.570 (0.252–1.288)		0.441 (0.184–1.060)		1.217 (0.833–1.777)		1.169 (0.791–1.728)	
Chemotherapy		0.501		0.072		0.801		0.656
Yes	Ref		Ref		Ref		Ref	
No	1.426 (0.508–4.007)		2.888 (0.911–9.157)		1.045 (0.743–0.469)		1.085 (0.758–1.553)	
Age	1.013 (0.987–1.040	0.329	1.014 (0.987–1.042)	0.323	1.012 (1.001–1.023)	0.038	1.013 (1.002–1.025)	0.024
Sex		0.668		0.704		0.621		0.510
Male	Ref		Ref		Ref		Ref	
Female	0.873 (0.469–1.624)		0.884 (0.469–1.667)		0.941 (0.740–1.197)		0.921 (0.722–1.176)	
Emergency surgery		0.209		0.353		0.073		0.082
No	Ref		Ref		Ref		Ref	
Yes	3.572 (0.490–26.065)		2.693 (0.334–21.744)		3.612 (0.888–14.693)		3.579 (0.852–15.031)	
Anastomotic leakage		0.114		0.041		0.000		0.000
No	Ref		Ref		Ref		Ref	
Yes	4.977 (0.680–36.435)		8.735 (1.090–70.000)		6.036 (2.223–16.392)		8.004 (2.855–22.438)	
Grade ^a^		0.053		NA		0.155		NA
I–II	Ref		Ref		NA		Ref	
III–IV and mucinous	2.360 (0.988–5.638)		NA		1.385 (0.885–2.167)		NA	
Lymphovascular invasion		0.392		0.703		0.649		0.985
No	Ref		Ref		Ref		Ref	
Yes	0.738 (0.369–1.478		0.862 (0.403–1.844)		0.943 (0.732–1.215)		1.003 (0.759–1.325)	
Perineural invasion		0.836		0.803		0.767		0.436
No	Ref		Ref		Ref		Ref	
Yes	1.090 (0.482–2.464)		0.891 (0.361–2.202)		0.952 (0.687–1.319)		0.865 (0.601–1.245)	

*HR* hazard ratio, *95% CI* 95% confidence interval^a^Eight patients with their primary tumors resected were excluded.

## Discussion

This 10-year follow-up study confirmed that patients with stage IIB/C cancer (pT4N0M0) had inferior oncological survival than did patients with stage IIIA cancer (pT1-2N1M0) with respect to 10-year locoregional recurrence-free survival and 10-year cancer-specific survival, with stage IIC (pT4bN0M0) showing the worst survival outcome. This staging paradox could not be solely explained by factors such as radicality, harvested lymph node, and chemotherapy administration. In addition, our study showed that the initial locoregional recurrence rate was higher in stage IIB/C (pT4N0M0) than in stage IIIA (pT1-2N1M0) cancer. Hence, stage IIB/C, particularly stage IIC (pT4bN0M0), is a subgroup of colon cancer that requires intense cytotoxic chemotherapy specific to its recurrence pattern to improve oncological outcomes.

Aberrant oncological outcomes of stage IIB/C colon cancer have received sustained attention; however, they remain to be elucidated. Numerous hypothesized explanations for the staging paradox focus on the insufficient treatment of stage IIB/C cancer, rather than on its substantially aggressive cancer behavior. Based on this hypothesis, patients with stage IIB/C cancer will demonstrate superior survival outcomes compared with those with stage IIIA cancer if sufficient treatment is provided. The proposed insufficient treatments include inadequate radicality and harvested lymph node and improper chemotherapy administration in patients with stage IIB/C compared with those with stage IIIA cancer [4]. Considering this rationale, the staging paradox disappears when these factors are eliminated.

Inadequate radicality has long been considered a risk factor for the paradox between stages IIB/C and IIIA. According to Chu et al., a positive margin could induce inferior survival outcomes in patients with stage IIB/C disease [6]. However, two other studies questioned this finding [12, 14]. Kim et al. and Shimizu et al. recruited patients with colon cancer who underwent R0 resection and reported that the staging paradox persisted. Our study also showed that the staging paradox persisted in patients with stage IIB/C colon cancer after adjusting for radicality.

Similarly, inadequate lymph node retrieval is believed to contribute to the misclassification of pT4N1 as pT4N0, resulting in understaging and insufficient adjuvant chemotherapy [4]. Nevertheless, emerging studies have opposed this idea [7, 14], demonstrating that patients with stage IIB/C colon cancer with harvested lymph nodes ≥ 12 did not exhibit better survival [15]. Our findings also suggested that the staging paradox persisted in patients with harvested lymph nodes ≥ 12.

The NCCN guidelines recommend adjuvant chemotherapy as the standard treatment for colon cancer with high-risk features, such as pT4 [1, 2]. Adjuvant chemotherapy administration is assumed to improve the survival outcomes of patients with pT4 colon cancer to a level similar to the outcomes of patients with stage III colon cancer [9]. However, the extent to which adjuvant chemotherapy prolongs survival remains unclear [2]. The effectiveness of adjuvant chemotherapy for stage IIB/C colon cancer may be related to many factors, such as the regimens used, course of chemotherapy, and completeness of chemotherapy. For instance, compliance with these guidelines remains low according to a national evaluation of adjuvant chemotherapy in patients with pT4N0M0 disease using the US National Cancer Database [11]. Our study also confirmed a low compliance rate with adjuvant chemotherapy for stage IIB/C colon cancer in our cohort.

The current NCCN guidelines recommend fluoropyrimidine- or oxaliplatin-based chemotherapy for patients with pT4N0M0 colon cancer [16]. Our study found that chemotherapy administration did not affect the oncological outcomes of pT4N0M0 colon cancer and that the administration of an oxaliplatin-based regimen did not also affect the oncological outcomes of patients with pT4N0M0 colon cancer. We believe that the sample size of patients with pT4N0M0 disease was significantly small to achieve sufficient statistical power to demonstrate the oncological benefits of adjuvant chemotherapy in patients with pT4N0M0 colon cancer.

Our study demonstrated that the rates of initial locoregional recurrence and peritoneal carcinomatosis were higher in patients with stage IIB/C colon cancer than in those with stage IIIA colon cancer, which is consistent with the findings of previous studies [10, 12, 14]. In addition, the incidence rate of peritoneal carcinomatosis was higher for patients with stage IIB colon cancer than for patients with stage IIC and IIIA colon cancers, a result concordant with that of a population-based study [17]. This recurrence pattern may reflect different routes of colon cancer cell spread. Stage IIB/C is a condition in which the tumor penetrates the visceral serosa and may cause tumor seeding over the peritoneum [17]. The potential of pathological N1c to describe a condition in which nearby lymph nodes do not contain cancer, but have cancer cells in tissues near the tumor, cannot be neglected. However, this hypothesis warrants further investigation.

We hypothesized that pT4N0M0 requires intense adjuvant chemotherapy, based on its recurrence patterns. If there is a predominance of locoregional recurrence, it may be more reasonable to consider intraperitoneal spread and chemotherapy. If systemic recurrence is predominant, systemic spread and chemotherapy should be considered.

Hyperthermic intraperitoneal chemotherapy (HIPEC) has been used to treat peritoneal carcinomatosis in certain malignancies, such as ovarian cancer [18]. Intra-abdominal administration of chemotherapy may theoretically be effective in eliminating peritoneally shed tumor cells, especially in patients with stage IIB/C colon cancer. However, the role of HIPEC in reducing peritoneal recurrence remains unclear. Although some randomized studies support the finding that HIPEC can improve locoregional control [19, 20], others have opposed the beneficial role of HIPEC in locally advanced colorectal cancer [21, 22].

Stage IIBC colon cancer exhibits aggressive behavior, indicating a different molecular signature of colon cancer [12]. This speculation is based on the observation of larger tumor size, higher CEA levels, and worse differentiation. A previous study by Kim et al. reported that pro-tumor inflammatory reactions were more prominent in stage II/C colon cancer [12]. Nevertheless, additional data should be collected to elucidate the differences between stage IIB/C and stage IIIA colon cancers.

This study has some limitations. First, the major limitation of this study was the small sample size. Increasing the sample size or considering multicenter data collection may enhance the robustness of this study. Second, this was a retrospective analysis, and inherent biases, such as selection and recall biases, could not be neglected. Third, the number of patients who received adjuvant chemotherapy, including oxaliplatin, was small. Adjuvant chemotherapy with oxaliplatin requires recruitment of more patients to evaluate its efficacy. Fourth, biomolecular markers, including mismatch repair, microsatellite instability, and v-raf murine sarcoma viral oncogene homolog B1, were not completely assessed in this study. Data were difficult to collect from the retrospective cohort of a previously maintained database. Fifth, the heterogeneity of chemotherapy regimens was used in our cohort, including oral metronomic uracil–tegafur (UFUR^®^), capecitabine (Xeloda^®^), or intravenous 5-fluorouracil- or oxaliplatin-based chemotherapy, which may hinder, to some extent, the interpretation of the effect of chemotherapy on the staging paradox.

In conclusion, pT4N0 colon cancer has inferior oncological survival in terms of 10-year locoregional recurrence-free survival and cancer-specific survival compared with pT1-2N1 colon cancer, which cannot be explained merely by radicality, harvested lymph nodes, and chemotherapy administration. The higher peritoneal recurrence rate for pT4N0M0 than for pT1-2N1M0 implies a possible intra-abdominal spreading pattern of pT4 colon cancer even after R0 resection. This study highlights the need for intensive cytotoxic chemotherapy to treat this recurrence pattern in patients with pT4 colon cancer.

## Data Availability

No datasets were generated or analysed during the current study.
